# Ba_3_Al_2_B_12_O_24_: a beryllium-free member of the Sr_2_Be_2_B_2_O_7_ family with a ^2^_∞_[B_12_Al_2_O_28_] double-layered structure

**DOI:** 10.1039/d5sc03854e

**Published:** 2025-09-04

**Authors:** Xiaorong Liu, Hongping Wu, Zhanggui Hu, Jiyang Wang, Yicheng Wu, Hongwei Yu

**Affiliations:** a State Key Laboratory of Crystal Materials, Tianjin Key Laboratory of Functional Crystal Materials, Institute of Functional Crystals, Tianjin University of Technology Tianjin 300384 China yuhw@email.tjut.edu.cn wuhp2022@163.com

## Abstract

Nonlinear optical (NLO) crystals capable of expanding the spectral region of solid-state are of great importance for many high-tech applications, yet their rational structure design remains a great challenge because of the conflicting property requirements among second harmonic generation (SHG) response, ultraviolet (UV) cut-off edge, and birefringence. Herein, based on the chemical disubstitution of the classic NLO crystal Sr_2_Be_2_B_2_O_7_ (SBBO), *i.e.*, substituting [BO_3_] triangles with larger π-conjugated [B_3_O_7_] groups and substituting high-toxic [BeO_4_] tetrahedra with environment-friendly [AlO_4_] tetrahedra, a new high-performance aluminoborate NLO crystal, Ba_3_Al_2_B_12_O_24_, has been successfully designed and synthesized. The theoretical calculations and optical property measurements indicate that Ba_3_Al_2_B_12_O_24_ exhibits not only the largest SHG response among the reported aluminoborates (2.7 × KH_2_PO_4_), but also a short UV absorption edge (<190 nm) and moderate birefringence. More importantly, Ba_3_Al_2_B_12_O_24_ melts congruently, and a single crystal with a size of 9 × 5 × 2 mm^3^ has been grown by the top-seeded-solution-growth method. These demonstrate that Ba_3_Al_2_B_12_O_24_ is a promising NLO crystal and the chemical disubstitution is a feasible strategy for the exploration of functional materials.

## Abstract 2

A new Sr_2_Be_2_B_2_O_7_-like aluminoborate Ba_3_Al_2_B_12_O_24_ was synthesized, satisfying performance balance between large SHG response, a short cutoff edge, and appropriate birefringence.

## Graphical abstract

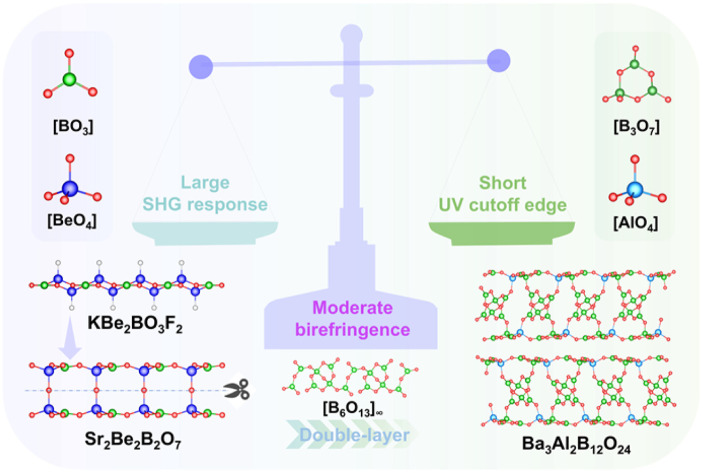

## Introduction

Nonlinear optical (NLO) materials, as the core components of all-solid-state lasers, play a crucial role in current scientific and technological applications.^1–8^ Based on these NLO crystals, ultraviolet (UV) and deep-ultraviolet (DUV; *λ* < 200 nm) coherent radiation can be directly obtained through cascading second harmonic generation (SHG), which has a variety of applications in laser micromachining, semiconductor manufacturing, photolithography, and high-resolution spectroscopy.^9–15^

During the past few decades, borates have been considered a major part of the exploration of UV or DUV NLO crystals because of their excellent structural units, *i.e.*, [BO_3_] or [BO_4_], and their combinations subsequently result in the formation of a wide variety of structural topologies,^16–18^ such as [BO_3_] in KBe_2_BO_3_F_2_ (KBBF),^19^ [B_3_O_6_] in β-BaB_2_O_4_ (β-BBO),^20^ [B_3_O_7_] in LiB_3_O_5_ (LBO),^21^ and [B_3_O_7_] in CsLiB_6_O_10_ (CLBO).^22^ These functional units make borate possess not only a short UV cutoff edge, but also a large SHG response in non-centrosymmetric (NCS) structures through their excellent combination and arrangement. Among them, in KBBF, [BO_3_] groups with a coplanar configuration and an aligned arrangement are linked by [BeO_3_F] to build unique ^2^_∞_[Be_2_BO_3_F_2_] layers, which result in the balance of its short cutoff edge, suitable SHG response and birefringence. These superior optical properties make KBBF the sole NLO crystal to generate DUV coherent radiation light by SHG. However, its strong layering tendency due to the weak interlayer bonding and the high toxicity of the contained beryllium limits the wide applications of KBBF in the DUV region.^19^ To overcome the layer habit of KBBF, an effective strategy is to develop borates with reinforced interlayer bonding by introducing stronger B–O/Be–F bonds or developing single layers into double layers.^23^ The successful examples include NaSr_3_Be_3_B_3_O_9_F_4_ (NSBBF),^24^ Na_2_CsBe_6_B_5_O_15_ (NCBBO),^25^ Sr_2_Be_2_B_2_O_7_ (SBBO),^26^*etc.* However, the toxicity of beryllium still hinders their applications.^27^

Aluminoborates have been considered attractive candidates for replacing Be-borates or Be-borate fluorides in UV NLO materials since the tetrahedral coordination environment of the Al^3+^ cation is similar to that of the Be^2+^ cation, and aluminum has lower toxicity than beryllium.^28^ Based on this, a series of aluminoborates have been synthesized, such as K_2_Al_2_B_2_O_7_,^29,30^ β-Rb_2_Al_2_B_2_O_7_,^28^ Cs_2_Al_2_B_6_O_13_,^31^ K_3_Ba_3_Li_2_Al_4_B_6_O_20_F,^32^ Rb_3_Ba_3_Li_2_Al_4_B_6_O_20_F,^33^*etc.* Among them, K_2_Al_2_B_2_O_7_ has a transparent window into the DUV region (180–3600 nm) and an appropriate birefringence of Δ*n* = 0.07@589 nm. The shortest Type I phase-matching (PM) wavelength is evaluated to be 232.5 nm, which can be used for the fourth harmonic generation (FHG) of the Nd:YAG laser (1064 nm).^34^ The results demonstrate the UV NLO application potential of K_2_Al_2_B_2_O_7_. As such, we will continue to explore new UV or DUV NLO crystals in aluminoborates.

Through systematic investigations, a new beryllium-free SBBO-like aluminoborate, Ba_3_Al_2_B_12_O_24_, was successfully synthesized. In Ba_3_Al_2_B_12_O_24_, the two-dimensional (2D) ^2^_∞_[B_3_AlO_6_] single layers are derived from the ^2^_∞_[Be(BO_3_)O] single layers of SBBO, in which the [BeO_4_] tetrahedra are replaced by [AlO_4_] tetrahedra and the [BO_3_] triangles are substituted by [B_3_O_7_] units. The single layers are linked by the one-dimensional (1D) ^1^_∞_[B_6_O_13_] chains, forming ^2^_∞_[B_12_Al_2_O_28_] double layers with tunnels, which are filled with the Ba^2+^ cations. Meanwhile, Ba_3_Al_2_B_12_O_24_ exhibits a large SHG response (2.7 × KH_2_PO_4_ (KDP)), a short absorption edge (<190 nm), and suitable birefringence. Compared to other reported aluminoborates, Ba_3_Al_2_B_12_O_24_ achieves an optimal balance between SHG efficiency and DUV transparency. Notably, congruently melting thermal properties are also observed, and a single crystal with 9 × 5 × 2 mm^3^ has been grown by a top-seeded-solution-growth (TSSG) technique. These results demonstrate that Ba_3_Al_2_B_12_O_24_ is a promising UV NLO material. In this report, we present the synthesis, crystal structure, thermal behavior, NLO properties, structure–property relationships, and the first-principles calculations of Ba_3_Al_2_B_12_O_24_.

## Results and discussion

Pure polycrystalline Ba_3_Al_2_B_12_O_24_ was synthesized through a high-temperature solid-state reaction. The experimental powder X-ray diffraction (PXRD) patterns exhibit good agreement with the calculated results derived from the structural data (Fig. S1). To investigate the thermal behavior of Ba_3_Al_2_B_12_O_24_, thermogravimetry-differential scanning calorimetry (TG-DSC) analysis was performed on the pure polycrystalline sample (Fig. S2). Clearly, there is only one endothermic peak at 840 °C on its DSC curve, and no weight loss is observed on the TG curve. To further confirm its thermal behavior, Ba_3_Al_2_B_12_O_24_ was heated to 850 °C, and the polycrystalline powder sample completely melted. Then the melt was slowly cooled to room temperature. The PXRD patterns of the sample after melting were consistent with those of the pure polycrystalline sample (Fig. S3). These results demonstrate that Ba_3_Al_2_B_12_O_24_ melts congruently. Owing to its congruent-melting behavior, a single crystal of Ba_3_Al_2_B_12_O_24_ with a size of 9 × 5 × 2 mm^3^ has also been successfully grown from its stoichiometric melt (Fig. S4).

**Fig. 1:**
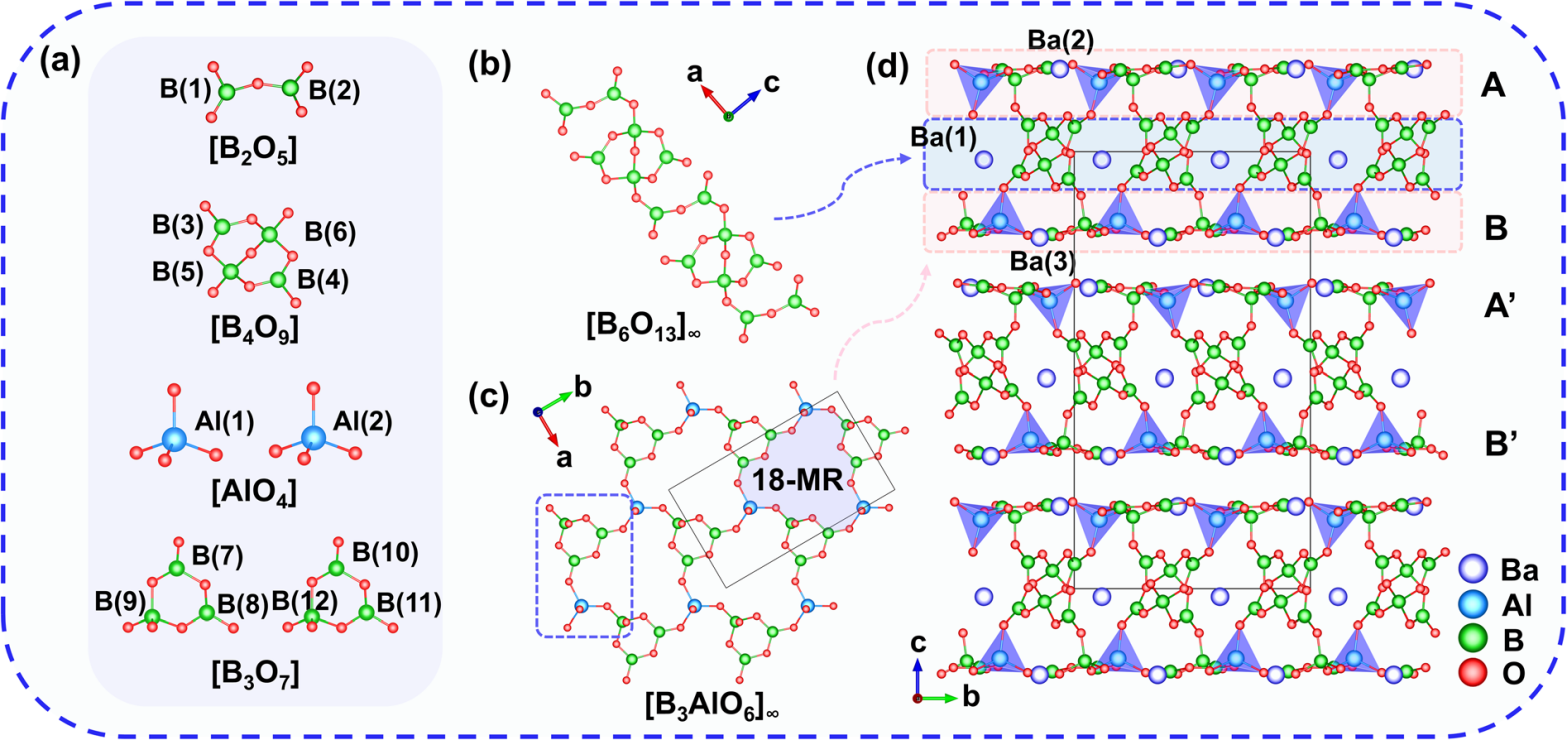
(a) Basic building units (BBUs) of Ba_3_Al_2_B_12_O_24_: [B_2_O_5_], [B_4_O_9_], [AlO_4_], and [B_3_O_7_] units; (b) ^1^_∞_[B_6_O_13_] chain of Ba_3_Al_2_B_12_O_24_; (c) ^2^_∞_[B_3_AlO_6_] layer of Ba_3_Al_2_B_12_O_24_; (d) 2D double-layered structure of Ba_3_Al_2_B_12_O_24_.

The structure of Ba_3_Al_2_B_12_O_24_ was determined by single-crystal X-ray diffraction (Table S1), which shows that Ba_3_Al_2_B_12_O_24_ crystallizes in the monoclinic space group of *Cc* (No. 9). The asymmetric unit of Ba_3_Al_2_B_12_O_24_ contains three unique Ba, two unique Al, twelve unique B, and twenty-four unique O atoms (Table S2). All of the Al atoms are four-coordinated, forming [AlO_4_] tetrahedra. The B atoms exhibit two different coordination environments: [BO_3_] triangles and [BO_4_] tetrahedra, which are further connected *via* corner-sharing to form a [B_2_O_5_] dimer, a [B_4_O_9_] group, and two types of [B_3_O_7_] groups with different symmetries (Fig. 1a). The [B_2_O_5_] dimers alternately connect with the [B_4_O_9_] groups to form a ^1^_∞_[B_6_O_13_] chain (Fig. 1b). The [B_3_O_7_] rings are linked with the [AlO_4_] tetrahedra to create ^2^_∞_[B_3_AlO_6_] layers, namely A, B, A′, and B′ (Fig. 1c). The arrangement of the ^2^_∞_[B_3_AlO_6_] single layers in Ba_3_Al_2_B_12_O_24_ is ABA′B′ABA′B′…, in which the A′ (B′) layers and the A (B) layers have a mirror image relationship along the *c*-axis. Furthermore, the A (A′) and B (B′) layers are connected by ^1^_∞_[B_6_O_13_] chains to generate ^2^_∞_[B_12_Al_2_O_28_] double layers (Fig. 1d). These layers are separated by the Ba^2+^ cations. Meanwhile, Ba^2+^ cations also occupy the void space within the double layer. The Ba atoms are in sixfold and tenfold coordination environments, [Ba(1)O_6_], [Ba(2)O_10_], and [Ba(3)O_10_] polyhedra. In the structure, the B–O distances fall in the range of 1.336(16) to 1.538(18) Å. The Al–O distances range from 1.728(9) to 1.780(9) Å. The Ba–O distances have a range from 2.593(9) to 3.133(9) Å (Table S3). The bond valence sum (BVS) analysis shows that the BVS values of Ba, Al, B, and O are 1.81–2.27, 3.08–3.13, 2.90–3.10, and 1.81–2.17, respectively (Table S2),^35^ which are in agreement with their ideal oxidation states for each atom.

**Fig. 2:**
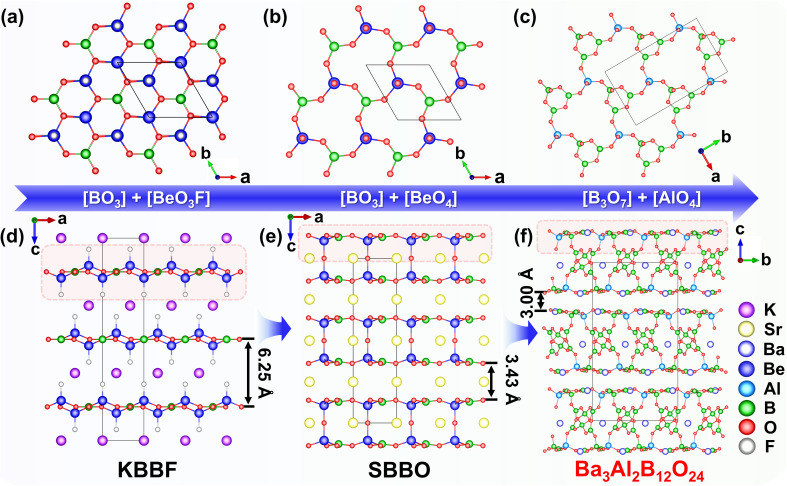
(a)–(c) Layers in KBBF, SBBO, and Ba_3_Al_2_B_12_O_24_, respectively; (d)–(f) the structural evolution from the single-layer structure of KBBF to the double-layer structures of SBBO and Ba_3_Al_2_B_12_O_24_.

From the above structural analysis, Ba_3_Al_2_B_12_O_24_ possesses a similar layered structure to KBBF (single-layer structure) and SBBO (double-layer structures). Therefore, we conducted their structure comparison (Fig. 2). All these crystals feature layered structural units composed of coplanar [BO_3_] triangles ([B_3_O_7_] for Ba_3_Al_2_B_12_O_24_) and tetrahedra ([BeO_3_F] for KBBF, [BeO_4_] for SBBO, and [AlO_4_] for Ba_3_Al_2_B_12_O_24_) (Fig. 2a–c). First, the [BeO_4_] tetrahedra were used to substitute the [BeO_3_F] tetrahedra in ^2^_∞_[Be_2_BO_3_F] single layers of KBBF, generating ^2^_∞_[Be(BO_3_)O] single layers in SBBO. Furthermore, the [BeO_4_] tetrahedra and the [BO_3_] units in the ^2^_∞_[Be(BO_3_)O] single layers of SBBO were replaced by [AlO_4_] tetrahedra and the [B_3_O_7_] units to form ^2^_∞_[B_3_AlO_6_] single layers of Ba_3_Al_2_B_12_O_24_. In SBBO, the ^2^_∞_[Be_2_(BO_3_)_2_O] double layers are formed by the ^2^_∞_[Be(BO_3_)O] single layers through bridged O atoms. Notably, the bridged O atoms between ^2^_∞_[Be(BO_3_)O] single layers are replaced by ^1^_∞_[B_6_O_13_] chains in the ^2^_∞_[B_12_Al_2_O_28_] double layers of Ba_3_Al_2_B_12_O_24_. Meanwhile, compared with KBBF, the weak interlayer bonding (K^+^–F^−^ ionic bonds) is evidently reinforced in the other two compounds (Sr–O bonds for SBBO and Ba–O bonds for Ba_3_Al_2_B_12_O_24_). In particular, the interlayer spacing greatly decreases from 6.25 Å in KBBF to 3.43 Å in SBBO and 3.00 Å in Ba_3_Al_2_B_12_O_24_ (Fig. 2d–f).^25,36^ The reduced interlayer space in Ba_3_Al_2_B_12_O_24_ is expected to reinforce the bonding force and will be conducive to overcoming the layering growth tendency. In Ba_3_Al_2_B_12_O_24_, the use of highly toxic beryllium oxide is effectively avoided by the substitution of the [BeO_4_] tetrahedra with the [AlO_4_] tetrahedra. Meanwhile, the structure contains planar [B_3_O_7_] groups with greater microscopic polarizability than the [BO_3_] groups, which can be conducive to generating large SHG response and optical anisotropy.

**Fig. 3:**
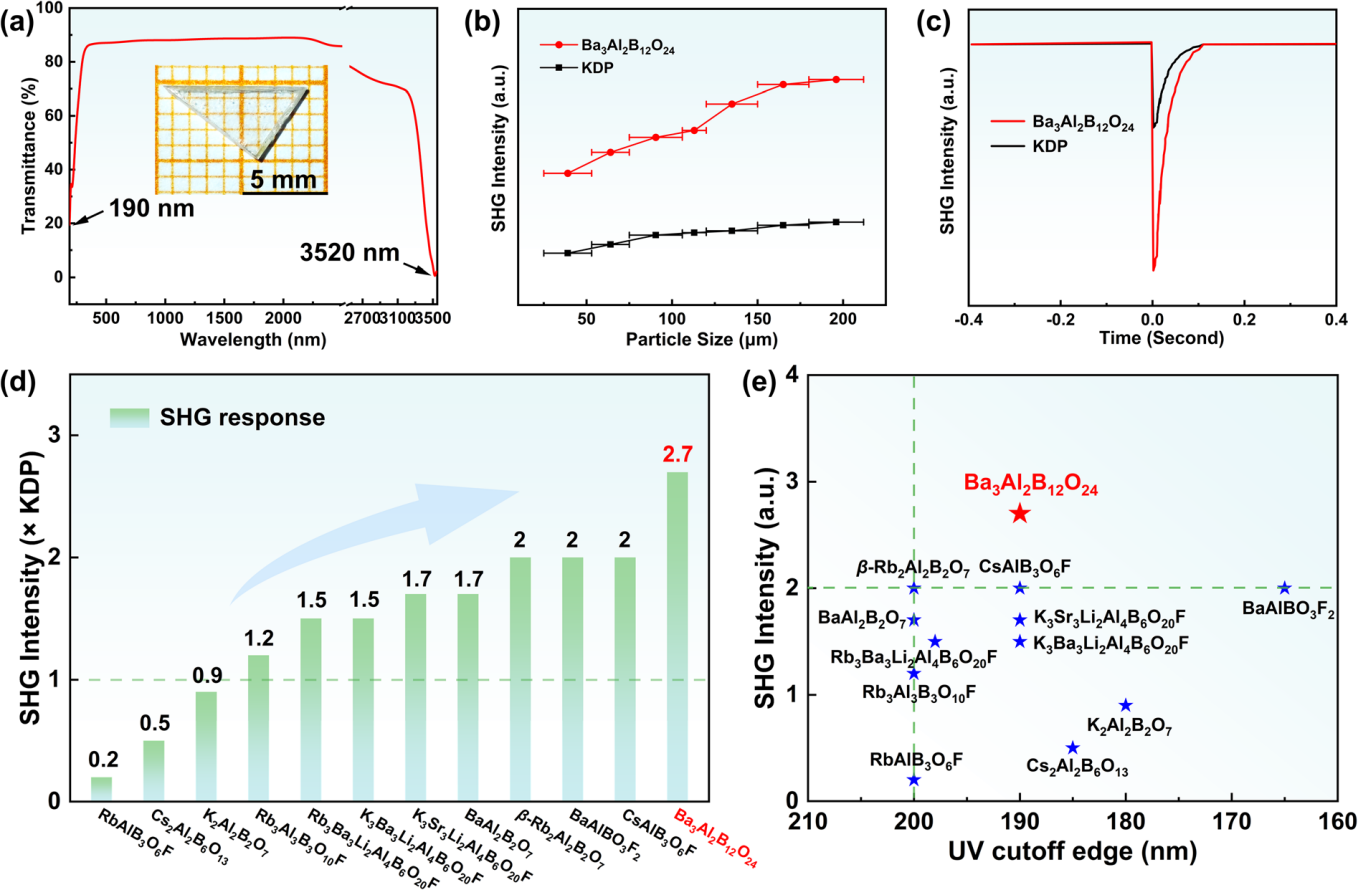
(a) Ultraviolet-visible and infrared (UV-Vis-IR) transmission spectra of single crystals of Ba_3_Al_2_B_12_O_24_; (b) and (c) powder SHG measurements at 1064 nm; (d) comparison of SHG responses of Ba_3_Al_2_B_12_O_24_ and reported aluminoborates; (e) scatter diagrams of SHG intensity and UV cutoff edges of Ba_3_Al_2_B_12_O_24_ and reported aluminoborates.

For Ba_3_Al_2_B_12_O_24_, a 9 × 5 × 2 mm^3^ crystal has been successfully obtained. The crystal can be further used to evaluate its optical properties. We measured its transparent windows from the UV to IR regions by using a polished Ba_3_Al_2_B_12_O_24_ crystal wafer with 2 mm thickness, which demonstrates that Ba_3_Al_2_B_12_O_24_ has a wide transparent spectral region from 190 to 3520 nm (Fig. 3a). This indicates that Ba_3_Al_2_B_12_O_24_ is DUV transparent, and its UV absorption edge is comparable with those of other aluminoborate materials, such as K_2_Al_2_B_2_O_7_ (180 nm),^30^ β-Rb_2_Al_2_B_2_O_7_ (<200 nm),^28^ Cs_2_Al_2_B_6_O_13_ (185 nm),^31^ K_3_Ba_3_Li_2_Al_4_B_6_O_20_F (190 nm),^32^ Rb_3_Ba_3_Li_2_Al_4_B_6_O_20_F (198 nm),^33^*etc.* As Ba_3_Al_2_B_12_O_24_ crystallizes in the NCS space group, powder SHG was measured using the Kurtz–Perry method. SHG intensity *versus* particle size revealed that Ba_3_Al_2_B_12_O_24_ is phase-matchable at 1064 nm and has a large SHG intensity, ∼2.7 × KDP (Fig. 3b and c). Furthermore, we investigated 11 reported NCS aluminoborate compounds containing alkali or alkaline earth metals (Table S4). When compared with other aluminoborates, Ba_3_Al_2_B_12_O_24_ exhibited the largest SHG response among these reported aluminoborates (Fig. 3d). As expected, Ba_3_Al_2_B_12_O_24_ demonstrates a good balance between SHG response and DUV transparency in aluminoborates (Fig. 3e).

**Fig. 4:**
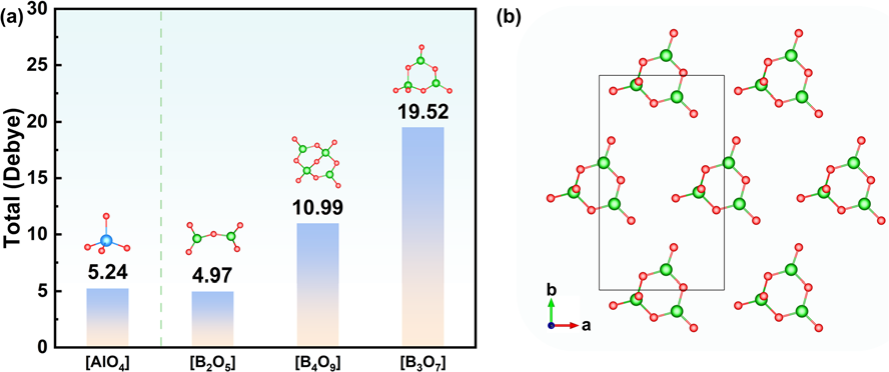
(a) The sum of the dipole moments of all corresponding units [AlO_4_], [B_2_O_5_], [B_4_O_9_], and [B_3_O_7_] in the unit cell of Ba_3_Al_2_B_12_O_24_; (b) the arrangements of [B_3_O_7_] groups in Ba_3_Al_2_B_12_O_24_.

In order to investigate the origin of the large SHG response of Ba_3_Al_2_B_12_O_24_, the dipole moment was calculated based on the BVS method to evaluate the contributions of individual groups to the SHG response (Table S5).^37^ We found that the dipole moments of the B–O anionic groups in Ba_3_Al_2_B_12_O_24_ are significantly larger than that of the Al–O anionic groups (Fig. 4a). This result indicates that the B–O anionic groups are important contributors to the large SHG response of Ba_3_Al_2_B_12_O_24_. Meanwhile, the [B_3_O_7_] units in the ^2^_∞_[B_3_AlO_6_] layers maintain an almost consistent coplanar arrangement, which is conducive to producing a large SHG response (Fig. 4b).

Besides these, birefringence is also important for NLO crystals to achieve a wide PM range. Therefore, the birefringence value of Ba_3_Al_2_B_12_O_24_ was measured using a cross-polarizing microscope based on the formula *R* = Δ*n* × *d*, where *R*, Δ*n*, and *d* are retardation, birefringence, and thickness of the crystal, respectively.^38^ In the measurement, the crystal of Ba_3_Al_2_B_12_O_24_ with a thickness of 18.7 μm was used. And the I-order green interference color was observed using a cross-polarized light microscope (Fig. S5b). By comparing with the Michal–Levy chart, *R* is approximately 780 nm. Consequently, the birefringence of Ba_3_Al_2_B_12_O_24_ is about 0.042 in the visible region. The birefringence of Ba_3_Al_2_B_12_O_24_ was calculated based on the formulae *ε*(*ω*) = *ε*_1_(*ω*) + i*ε*_2_(*ω*) and *n*^2^(*ω*) = *ε*(*ω*). The calculated birefringence of Ba_3_Al_2_B_12_O_24_ at 1064 nm is 0.041, which is close to the measured birefringence (Fig. S5a).

**Fig. 5:**
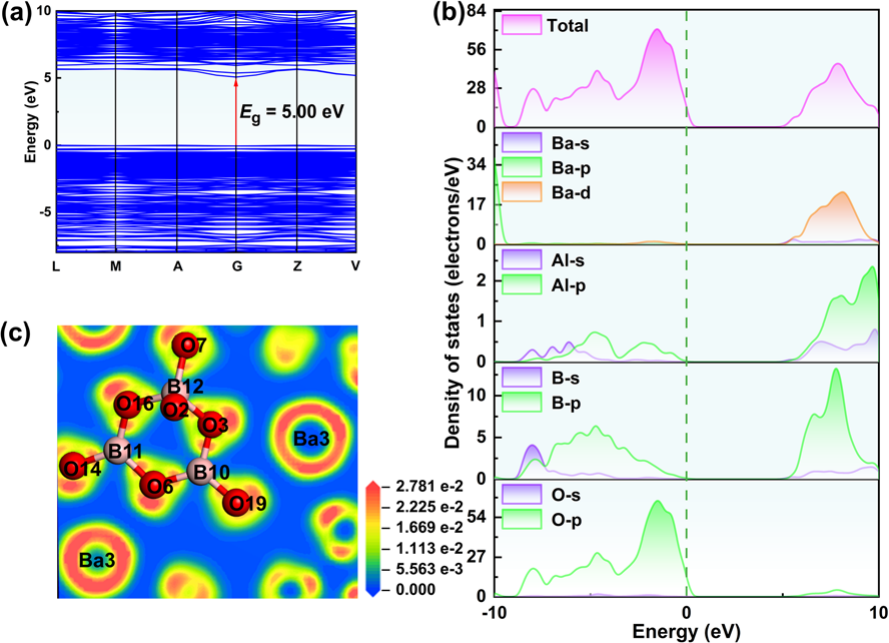
(a) Electronic band structure of Ba_3_Al_2_B_12_O_24_; (b) DOS and PDOS projected on the constitutional atoms of Ba_3_Al_2_B_12_O_24_; (c) ELF map of Ba_3_Al_2_B_12_O_24_.

To further explore the relationship between the structure and optical properties, the electronic structure was calculated *via* first-principles calculations based on the density functional theory (DFT).^39^ Ba_3_Al_2_B_12_O_24_ exhibits a direct band gap of 5.00 eV (Fig. 5a), which is smaller than the experimental result. This discrepancy can be attributed to the underestimation of the band gap (*E*_g_) using DFT calculations.^40^ Furthermore, we investigated the density of states (DOS) and partial density of states (PDOS) of Ba_3_Al_2_B_12_O_24_ (Fig. 5b). Since the optical properties of the compound are mainly determined by the states near the Fermi level,^41^ the top of the valence band (VB) and the bottom of the conduction band (CB) were analyzed. We found that the upper parts of the VB from −10 to 0 eV for Ba_3_Al_2_B_12_O_24_ mainly originate from the O 2p orbitals, which are mixed with a small part of B 2p orbitals and Al 3p orbitals. Meanwhile, the bottom of the CB from 0 to 10 eV primarily comprises B 2s2p orbitals with small amounts from the Ba 5d6s orbitals and Al 3s3p orbitals. From the above analysis, we can conclude that the large SHG response of Ba_3_Al_2_B_12_O_24_ is mainly attributable to the B–O anionic groups. Based on the electron localization function (ELF) maps (Fig. 5c), we can intuitively observe that the clover-shaped asymmetric electron densities are arranged around the oxygen atoms to form the NLO-active [B_3_O_7_] groups, which indicates that they have a positive contribution to the SHG response. Additionally, the nearby Ba cations form slightly distorted spherical asymmetric electron localized densities under the influence of the NLO-active [B_3_O_7_] groups, which also contributes to the SHG response (Fig. S6). Similar situations have also been found in other compounds, such as Cs_3_VO(O_2_)_2_CO_3_ and Ba_4_B_11_O_20_F.^42,43^ Therefore, the large SHG response in Ba_3_Al_2_B_12_O_24_ originates mainly from B–O groups.

## Conclusions

In summary, a new SBBO-like aluminoborate, Ba_3_Al_2_B_12_O_24_, has been successfully synthesized by the chemical disubstitution strategy. Ba_3_Al_2_B_12_O_24_ features ^2^_∞_[B_12_Al_2_O_28_] double layers composed of ^2^_∞_[B_3_AlO_6_] single layers that are transformed from ^2^_∞_[Be(BO_3_)O] single layers in SBBO through substituting [BeO_4_] tetrahedra and [BO_3_] triangles with [AlO_4_] tetrahedra and [B_3_O_7_] units, respectively, which are further linked together *via* interlayer ^1^_∞_[B_6_O_13_] chains. This connection enables the reduced interlayer space in Ba_3_Al_2_B_12_O_24_ to reinforce the bonding force and is conducive to overcoming the layering growth tendency. More importantly, Ba_3_Al_2_B_12_O_24_ satisfies the performance balance for NLO applications in the UV region among a large SHG response (2.7 × KDP), a short UV absorption edge (<190 nm), and a suitable birefringence. Structural analyses and theoretical calculations indicate that the [B_3_O_7_] units with coplanar configurations and well-ordered arrangements make a significant contribution to the excellent optical properties. Meanwhile, Ba_3_Al_2_B_12_O_24_ melts congruently, which is helpful for its crystal growth. These results demonstrate that site substitution based on the classical structure is an effective strategy for developing new UV and DUV NLO crystals.

## Author contributions

X. R. L. performed the experiments, data analysis, theoretical calculations, and paper writing. H. W. Y. designed and supervised the experiments. H. P. W. provided major revisions of the manuscript. Z. G. H. supervised the optical experiments. J. Y. W. and Y. C. W. helped with the analyses of the crystallization process and the data. All the authors discussed the results and commented on the manuscript.

## Conflicts of interest

There are no conflicts to declare.

## Supplementary Material

SC-016-D5SC03854E-s001

SC-016-D5SC03854E-s002

## Data Availability

CCDC 2480204 (Ba_3_Al_2_B_12_O_24_) contains the supplementary crystallographic data for this paper.^44^ The data supporting this article have been included as part of the SI. Supplementary information: Experimental section and additional tables and figures. See DOI: https://doi.org/10.1039/d5sc03854e.
