# Supplementary material for: Ba3Al2B12O24: a beryllium-free member of the Sr2Be2B2O7 family with a 2∞[B12Al2O28] double-layered structure
Source: Chem Sci. 2025 Sep 4;16(38):17766–71. doi: 10.1039/d5sc03854e (PMC12409481; doi:10.1039/d5sc03854e)
Supplement: SC-016-D5SC03854E-s001 [file SC-016-D5SC03854E-s001.pdf]

Supplementary Information (SI) for Chemical Science.
This journal is © The Royal Society of Chemistry 2025

Supporting Information

$Ba_3Al_2B_{12}O_{24}$: a beryllium-free member of the $Sr_2Be_2B_2O_7$ family with $^{2}_{\infty}[B_{12}Al_2O_{28}]$ double-layered structure

Xiaorong Liu,[a] Hongping Wu,*[a] Zhanggui Hu,[a] Jiyang Wang,[a] Yicheng Wu,[a] and Hongwei Yu*[a]

[a] *State Key Laboratory of Crystal Materials, Tianjin Key Laboratory of Functional Crystal Materials, Institute of Functional Crystals, Tianjin University of Technology, Tianjin 300384, China*

* *Corresponding author (email: yuhw@email.tjut.edu.cn; wuhp2022@163.com)*

CONTENTS

Experimental Section **S1**

Table S1 (Crystal data and structure refinement) **S5**

Table S2 (Atomic coordinates, displacement parameters, and BVS) **S6**

Table S3 (Bond lengths and angles) **S8**

Table S4 (Comparison of the properties of $Ba_3Al_2B_{12}O_{24}$ with the reported UV NLO aluminoborates) **S16**

Table S5 (The sum of the dipole moments and the normalized dipole moment of all corresponding units $[AlO_4]$, $[B_2O_5]$, $[B_4O_9]$, and $[B_3O_7]$ in the unit cell of $Ba_3Al_2B_{12}O_{24}$) **S17**

Figure S1 (Calculated and experimental PXRD patterns of $Ba_3Al_2B_{12}O_{24}$) **S18**

Figure S2 (The TG/DSC curves for $Ba_3Al_2B_{12}O_{24}$) **S19**

Figure S3 (The PXRD of $Ba_3Al_2B_{12}O_{24}$ before and after melting) **S20**

Figure S4 ($Ba_3Al_2B_{12}O_{24}$ with a size of $9 \times 5 \times 2$ mm^3) **S21**

Figure S5 (The calculated birefringence of $Ba_3Al_2B_{12}O_{24}$) **S22**

Figure S6 (The sliced-plane of electron localization function (ELF) of $Ba_3Al_2B_{12}O_{24}$ in the *bc*-plane) **S23**

References **S24**

Experimental Section

Reagents

All raw materials including $Ba(OH)_2•8H_2O$ (Aladdin Chemistry Co., Ltd., 98%), H_3BO_3 (Aladdin Chemistry Co., Ltd., 99%), Al_2O_3 (Tianjin Fu Chen Chemical Co., Ltd., 99%) were used as received from commercial sources without any further purification.

Solid-state synthesis

The polycrystalline powder samples of the $Ba_3Al_2B_{12}O_{24}$ were synthesized through the solid-state reaction method by mixing raw materials of 1.586 g (3.0 mmol) of $Ba(OH)_2•8H_2O$, 0.171 g (1.0 mmol) of Al_2O_3, and 1.243 g (12.0 mmol) of H_3BO_3. The starting reagents were mixed thoroughly and ground homogeneously in an agate mortar. Then, the mixture was loaded into a platinum crucible and preheated in a muffle furnace at 500 °C for 10 h to eliminate H_2O. After being fully ground again, the mixture was heated to 800 °C and kept for 96 h. The materials were ground between heating procedures. With this procedure, a pure polycrystalline sample of $Ba_3Al_2B_{12}O_{24}$ was successfully obtained. Then, the polycrystalline powder sample was heated to 900 °C and kept for 2 h, and the melt was cooled to was cooled to 750 °C at a rate of 5 °C/h and cooled to room temperature at a rate of 20 °C/h, the sample after melting was obtained.

Crystal growth

Single-crystals of $Ba_3Al_2B_{12}O_{24}$ were obtained by the high-temperature melt method. A mixture of 1.586 g (3.0 mmol) of $Ba(OH)_2•8H_2O$, 0.171 g (1.0 mmol) of Al_2O_3, and 1.243 g (12.0 mmol) of H_3BO_3 was loaded into a platinum crucible. The mixture was heated to 870 °C and kept for 6 h to form a homogeneous melt. Subsequently, the melt was cooled to 850 °C. Then the temperature was cooled to 750 °C at a rate of 2 °C/h and cooled to room temperature at a rate of 10 °C/h. In this way, a few millimeter-sized transparent block-shaped crystals were grown and separated from the platinum crucible for further characterization.

One of the crystals is taken as the seed crystal and the top-seeded-solution-growth (TSSG) method is adopted to grow a large-size single crystal. The mixture containing $Ba(OH)_2 \bullet 8H_2O$, Al_2O_3, and H_3BO_3 (37.001, 3.986, and 29.013 g) was weighed. Similar to the seed growth, the mixture was placed in a Φ 40 mm × 40 mm platinum crucible and heated to 840 °C for 12 h to obtain a homogeneous melt. In order to ensure the saturation temperature of $Ba_3Al_2B_{12}O_{24}$, the seed was fixed on a corundum rod with the aid of platinum wire and slowly immersed into the melt. Then, the saturation temperature of 817 °C was determined by observing the growth or dissolution of the seed. A $Ba_3Al_2B_{12}O_{24}$ seed was dipped into the surface of the solution at 3 °C higher than the saturation temperature (820 °C), followed by decreasing the temperature to the saturation point over 1 h. From the saturation temperature (817 °C), the solution was cooled to 815 °C at a rate of 0.1 °C per day, with the seed crystal rotated at 5 rpm. The crystal was removed from the solution, and then the temperature was cooled to 600 °C at a rate of 5 °C/h and subsequently cooled to room temperature at a rate of 20 °C/h. In the end, a $Ba_3Al_2B_{12}O_{24}$ crystal with dimensions of 9 × 5 × 2 mm^3 was obtained.

Powder X-ray diffraction

Powder X-ray diffraction (PXRD) data measurements were carried out on a Bruker D2 PHASER diffractometer equipped with Cu-Kα radiation at room temperature. All the data were collected in the 2θ range of 10°–70° with a step size of 0.01° and a step time of 2 s.

Single-crystal structure determination

A transparent single crystal of $Ba_3Al_2B_{12}O_{24}$ was determined by the single-crystal XRD. Diffraction data were collected at 273 K on a Bruker SMART APEX II 4K CCD diffractometer equipped with Mo-Kα radiation (λ = 0.71073 Å) and integrated with the SAINT program.[1] The structure was solved by direct methods using SHELXT and refined by full-matrix least-squares methods against F^2 by SHELXL-2019/1.[2] Finally, the reasonable Goodness-of-fit and R indices were obtained. The PLATON was used to check the symmetry of the structure, and no higher symmetries were found.[3] Crystallographic data, data collection, and structure refinement are summarized in Table S1. The atomic coordinates and equivalent isotropic displacement parameters,

bond valence sum (BVS) calculations for atoms, and selected bond lengths and angles are given in Tables S2–S3.

Thermal analysis

The thermal behavior of $Ba_3Al_2B_{12}O_{24}$ was measured using a NETZSCH STA 449F5 thermal analysis instrument (Germany) under flowing nitrogen gas. The sample was heated at a rate of 10 °C/min from 25 to 1000 °C in a platinum crucible and cooled down to 25 °C at a rate of 10 °C/min.

Optical transmittance spectra measurements

The UV and infrared (IR) transmission measurements were measured at room temperature using a Hitachi UV-Vis-NIR spectrophotometer (Japan) and a Nicolet iS50 Fourier transform infrared spectroscopy (FT-IR) spectrometer, with a transparent $Ba_3Al_2B_{12}O_{24}$ crystal used for the measurements.

SHG measurements

The SHG response of $Ba_3Al_2B_{12}O_{24}$ was evaluated using the Kurtz-Perry method with a Q-switched Nd:YAG laser at the wavelength of 1064 nm.[4] Powder samples were ground and sieved into distinct particle size ranges: 25–53, 53–75, 75–106, 106–120, 120–150, 150–180, and 180–212 μm, respectively. KDP samples with the same particle sizes were served as the references.

Birefringence measurements

The birefringence of $Ba_3Al_2B_{12}O_{24}$ was measured using a cross-polarizing microscope. On the basis of the crystal optics, the birefringence was calculated from the following formula: $R = \Delta n \times d$, where Δn is the birefringence, d is the thickness, and R is the retardation.

Theoretical calculations

The electronic structures and optical properties of $Ba_3Al_2B_{12}O_{24}$ were calculated by a plane-wave pseudopotential density functional theory (DFT) method employed by CASTEP software.[5] Perdew-Burke-Ernzerhof (PBE) functional with generalized gradient approximation (GGA) scheme was employed for all calculations, and the pseudopotential was set as a norm-conserving pseudopotential (NCP).[6] The following electrons were treated as valence electrons: Ba $5s^2 5p^6 6s^2$, Al $3s^2 3p^1$, B $2s^2 2p^1$, O $2s^2 2p^4$.

The plane-wave basis energy cutoff was set at 810.0 eV for $Ba_3Al_2B_{12}O_{24}$. The corresponding Monkhorst-Pack k-point meshes were adopted 5 × 3 × 1.[7]

Table S1. Crystal data and structure refinement for $Ba_3Al_2B_{12}O_{24}$.

	$\mathbf{Ba_3Al_2B_{12}O_{24}}$
Formula weight	979.70
Temperature	273(2) K
Wavelength	0.71073 Å
Crystal system	Monoclinic
Space group	*Cc* (No. 9)
Unit cell dimensions	a = 7.0957(10) Å<br>b = 12.1687(17) Å<br>c = 22.545(3) Å<br>β = 91.046(3) °
Volume	1946.4(5) Å^3
Z	4
Density	3.343 Mg/m^3
Absorption coefficient	6.220 mm^{-1}
F(000)	1784
Crystal size	0.066 × 0.045 × 0.041 mm^3
Theta range for data collection	3.324 to 27.596°
Index ranges	$-8<=h<=9$, $-15<=k<=15$, $-29<=l<=29$
Reflections collected	11765
Independent reflections	4367 [R(int) = 0.0486]
Completeness to theta = 25.242°	99.9 %
Refinement method	Full-matrix least-squares on F^2
Absolute structure parameter	0.11(3)
Data / restraints / parameters	4367 / 2 / 371
Goodness-of-fit on F^2	1.067
Final R indices [I>2sigma(I)][a]	R_1 = 0.0363, wR_2 = 0.0688
R indices (all data)	R_1 = 0.0442, wR_2 = 0.0733
Largest diff. peak and hole	0.983 and -0.992 eÅ^{-3}

[a] $R_1 = \Sigma||F_o| - |F_c||/\Sigma|F_o|$ and $wR_2 = [\Sigma w(F_o^2 - F_c^2)^2 / \Sigma w F_o^4]^{1/2}$ for $F_o^2 > 2\sigma(F_o^2)$

Table S2. Atomic coordinates and equivalent isotropic displacement parameters for $Ba_3Al_2B_{12}O_{24}$. U_{eq} is defined as one third of the trace of the orthogonalized U_{ij} tensor.

Atoms	*x*	*y*	*z*	U_{eq}	BVS
Ba (1)	0.17875(12)	0.88260(7)	0.48110(5)	0.0355(2)	1.81
Ba (2)	0.36494(9)	0.56104(6)	0.68784(4)	0.01733(16)	2.27
Ba (3)	0.09943(8)	0.64690(6)	0.30302(4)	0.01819(17)	2.12
Al (1)	-0.1310(5)	0.3944(3)	0.65820(17)	0.0158(8)	3.08
Al (2)	0.6127(5)	0.8147(3)	0.34028(19)	0.0177(8)	3.13
B (1)	0.5960(19)	0.6958(11)	0.5603(7)	0.017(3)	3.09
B (2)	0.733(2)	0.7345(13)	0.4547(7)	0.022(3)	3.10
B (3)	0.000(2)	0.4982(12)	0.5557(7)	0.022(3)	2.90
B (4)	0.315(2)	0.5236(13)	0.4376(7)	0.024(3)	3.05
B (5)	0.293(2)	0.5914(13)	0.5412(7)	0.019(3)	3.06
B (6)	0.043(2)	0.6253(12)	0.4700(6)	0.017(3)	2.95
B (7)	0.9120(19)	0.6127(12)	0.6911(6)	0.015(3)	3.04
B (8)	1.0025(19)	0.8048(12)	0.6853(7)	0.016(3)	3.06
B (9)	0.678(2)	0.7511(12)	0.6642(7)	0.019(3)	3.07
B (10)	0.6517(19)	0.5929(12)	0.3105(6)	0.016(3)	3.05
B (11)	0.739(2)	0.4011(12)	0.3204(7)	0.017(3)	3.02
B (12)	0.412(2)	0.4558(12)	0.3346(7)	0.017(3)	3.03
O (1)	1.0496(12)	0.6927(7)	0.6881(5)	0.020(2)	2.17
O (2)	0.4318(12)	0.4718(8)	0.3989(4)	0.025(2)	2.06
O (3)	0.4673(12)	0.5622(7)	0.3050(4)	0.0174(19)	2.05
O (4)	-0.0536(14)	0.5341(8)	0.4991(4)	0.024(2)	2.06
O (5)	-0.1242(12)	0.4295(8)	0.5837(4)	0.021(2)	2.08
O (6)	0.7878(12)	0.5120(7)	0.3197(5)	0.022(2)	2.15
O (7)	0.2289(12)	0.4275(7)	0.3121(4)	0.0164(18)	2.05
O (8)	0.8178(11)	0.8312(6)	0.6900(4)	0.0163(19)	2.12
O (9)	0.9695(11)	0.5065(7)	0.6985(4)	0.0168(18)	2.02

O (10)	0.4930(11)	0.7798(7)	0.6832(4)	0.0174(19)	2.05
O (11)	0.7276(11)	0.6405(7)	0.6878(4)	0.0155(18)	2.07
O (12)	0.3694(14)	0.5160(8)	0.4954(4)	0.024(2)	2.05
O (13)	0.1711(12)	0.5282(7)	0.5809(4)	0.0196(19)	1.99
O (14)	0.8728(13)	0.3224(7)	0.3224(5)	0.024(2)	2.02
O (15)	1.1323(12)	0.8820(7)	0.6759(4)	0.021(2)	2.07
O (16)	0.5537(12)	0.3713(7)	0.3172(4)	0.020(2)	2.06
O (17)	0.1565(13)	0.5782(8)	0.4184(4)	0.024(2)	1.92
O (18)	0.1805(12)	0.6766(7)	0.5105(4)	0.0198(19)	1.97
O (19)	0.7120(12)	0.6973(7)	0.3080(4)	0.020(2)	2.07
O (20)	0.6299(14)	0.7992(8)	0.4168(5)	0.031(2)	1.81
O (21)	0.6392(16)	0.6944(11)	0.5019(5)	0.046(3)	2.08
O (22)	0.9142(14)	0.7070(8)	0.4433(5)	0.026(2)	1.96
O (23)	0.7046(13)	0.7502(7)	0.6001(4)	0.021(2)	1.85
O (24)	0.4440(13)	0.6347(7)	0.5782(4)	0.0208(19)	2.13

Table S3. Bond lengths [Å] and angles [deg] for $Ba_3Al_2B_{12}O_{24}$.

Ba(1)-O(18)	2.593(9)	Ba(3)-O(10)#6	2.930(9)
Ba(1)-O(4)#1	2.673(9)	Ba(3)-O(8)#6	3.018(9)
Ba(1)-O(5)#1	2.741(9)	Ba(3)-O(9)#7	3.133(9)
Ba(1)-O(2)#2	2.750(9)	Ba(3)-B(10)#3	3.252(14)
Ba(1)-O(12)#2	2.754(10)	Ba(3)-B(11)#2	3.270(15)
Ba(1)-O(22)#3	2.959(10)	Al(1)-O(5)	1.735(10)
Ba(1)-B(3)#1	3.139(15)	Al(1)-O(10)#4	1.738(9)
Ba(1)-B(4)#2	3.235(17)	Al(1)-O(15)#8	1.740(10)
Ba(1)-B(6)	3.284(15)	Al(1)-O(9)#3	1.780(9)
Ba(2)-O(24)	2.697(9)	Al(2)-O(7)#1	1.728(9)
Ba(2)-O(11)	2.749(8)	Al(2)-O(20)	1.738(11)
Ba(2)-O(1)#3	2.752(9)	Al(2)-O(14)#2	1.745(10)
Ba(2)-O(13)	2.784(9)	Al(2)-O(19)	1.757(10)
Ba(2)-O(10)	2.815(9)	B(1)-O(23)	1.346(17)
Ba(2)-O(8)#4	2.817(8)	B(1)-O(21)	1.358(18)
Ba(2)-O(9)#3	2.898(8)	B(1)-O(24)	1.377(15)
Ba(2)-O(15)#4	2.904(9)	B(2)-O(21)	1.356(18)
Ba(2)-O(7)#5	2.984(9)	B(2)-O(22)	1.358(18)
Ba(2)-O(3)#5	3.111(9)	B(2)-O(20)	1.364(19)
Ba(2)-B(9)	3.257(14)	B(3)-O(5)	1.374(17)
Ba(2)-B(8)#4	3.268(14)	B(3)-O(13)	1.381(17)
Ba(3)-O(17)	2.755(9)	B(3)-O(4)	1.395(18)
Ba(3)-O(16)#2	2.769(9)	B(4)-O(12)	1.355(19)
Ba(3)-O(6)#3	2.785(9)	B(4)-O(2)	1.368(18)
Ba(3)-O(3)	2.806(9)	B(4)-O(17)	1.370(19)
Ba(3)-O(19)#3	2.821(9)	B(5)-O(24)	1.443(18)
Ba(3)-O(7)	2.830(8)	B(5)-O(13)	1.474(17)
Ba(3)-O(14)#2	2.912(9)	B(5)-O(18)	1.475(17)

B(5)-O(12)	1.489(17)	O(4)#1-Ba(1)-O(2)#2	105.8(3)
B(6)-O(18)	1.463(17)	O(5)#1-Ba(1)-O(2)#2	142.7(3)
B(6)-O(4)	1.466(16)	O(18)-Ba(1)-O(12)#2	122.7(3)
B(6)-O(22)#3	1.472(17)	O(4)#1-Ba(1)-O(12)#2	98.1(3)
B(6)-O(17)	1.538(18)	O(5)#1-Ba(1)-O(12)#2	100.0(3)
B(7)-O(11)	1.352(16)	O(2)#2-Ba(1)-O(12)#2	49.2(3)
B(7)-O(9)	1.364(16)	O(18)-Ba(1)-O(22)#3	51.4(3)
B(7)-O(1)	1.381(15)	O(4)#1-Ba(1)-O(22)#3	170.8(3)
B(8)-O(15)	1.336(16)	O(5)#1-Ba(1)-O(22)#3	134.8(3)
B(8)-O(8)	1.355(16)	O(2)#2-Ba(1)-O(22)#3	72.4(3)
B(8)-O(1)	1.405(16)	O(12)#2-Ba(1)-O(22)#3	87.5(3)
B(9)-O(10)	1.432(17)	O(18)-Ba(1)-B(3)#1	107.2(3)
B(9)-O(23)	1.460(18)	O(4)#1-Ba(1)-B(3)#1	26.2(3)
B(9)-O(11)	1.488(16)	O(5)#1-Ba(1)-B(3)#1	25.9(3)
B(9)-O(8)	1.501(16)	O(2)#2-Ba(1)-B(3)#1	129.2(3)
B(10)-O(19)	1.342(16)	O(12)#2-Ba(1)-B(3)#1	104.2(4)
B(10)-O(3)	1.364(16)	O(22)#3-Ba(1)-B(3)#1	158.1(3)
B(10)-O(6)	1.392(16)	O(18)-Ba(1)-B(4)#2	126.2(3)
B(11)-O(14)	1.348(16)	O(4)#1-Ba(1)-B(4)#2	103.9(4)
B(11)-O(16)	1.366(17)	O(5)#1-Ba(1)-B(4)#2	122.5(3)
B(11)-O(6)	1.392(17)	O(2)#2-Ba(1)-B(4)#2	24.8(4)
B(12)-O(7)	1.426(16)	O(12)#2-Ba(1)-B(4)#2	24.5(3)
B(12)-O(2)	1.468(17)	O(22)#3-Ba(1)-B(4)#2	78.3(3)
B(12)-O(16)	1.496(17)	B(3)#1-Ba(1)-B(4)#2	119.4(4)
B(12)-O(3)	1.512(16)	O(18)-Ba(1)-B(6)	25.5(3)
O(18)-Ba(1)-O(4)#1	128.9(3)	O(4)#1-Ba(1)-B(6)	151.1(3)
O(18)-Ba(1)-O(5)#1	89.2(3)	O(5)#1-Ba(1)-B(6)	114.0(3)
O(4)#1-Ba(1)-O(5)#1	51.5(3)	O(2)#2-Ba(1)-B(6)	98.2(3)
O(18)-Ba(1)-O(2)#2	123.6(3)	O(12)#2-Ba(1)-B(6)	109.7(3)

O(22)#3-Ba(1)-B(6)	26.6(3)	O(8)#4-Ba(2)-O(15)#4	48.3(2)
B(3)#1-Ba(1)-B(6)	132.6(4)	O(9)#3-Ba(2)-O(15)#4	118.1(2)
B(4)#2-Ba(1)-B(6)	104.6(4)	O(24)-Ba(2)-O(7)#5	157.1(2)
O(24)-Ba(2)-O(11)	70.9(3)	O(11)-Ba(2)-O(7)#5	107.6(3)
O(24)-Ba(2)-O(1)#3	89.5(3)	O(1)#3-Ba(2)-O(7)#5	72.2(3)
O(11)-Ba(2)-O(1)#3	123.8(3)	O(13)-Ba(2)-O(7)#5	131.1(2)
O(24)-Ba(2)-O(13)	50.3(3)	O(10)-Ba(2)-O(7)#5	95.8(2)
O(11)-Ba(2)-O(13)	119.9(3)	O(8)#4-Ba(2)-O(7)#5	89.4(3)
O(1)#3-Ba(2)-O(13)	72.3(3)	O(9)#3-Ba(2)-O(7)#5	66.6(2)
O(24)-Ba(2)-O(10)	65.1(3)	O(15)#4-Ba(2)-O(7)#5	110.2(3)
O(11)-Ba(2)-O(10)	50.6(2)	O(24)-Ba(2)-O(3)#5	152.7(3)
O(1)#3-Ba(2)-O(10)	73.3(2)	O(11)-Ba(2)-O(3)#5	88.0(3)
O(13)-Ba(2)-O(10)	105.0(3)	O(1)#3-Ba(2)-O(3)#5	117.1(3)
O(24)-Ba(2)-O(8)#4	111.9(3)	O(13)-Ba(2)-O(3)#5	140.4(2)
O(11)-Ba(2)-O(8)#4	117.4(2)	O(10)-Ba(2)-O(3)#5	114.6(2)
O(1)#3-Ba(2)-O(8)#4	118.8(2)	O(8)#4-Ba(2)-O(3)#5	62.1(2)
O(13)-Ba(2)-O(8)#4	79.4(3)	O(9)#3-Ba(2)-O(3)#5	91.8(2)
O(10)-Ba(2)-O(8)#4	167.9(2)	O(15)#4-Ba(2)-O(3)#5	64.8(3)
O(24)-Ba(2)-O(9)#3	111.7(3)	O(7)#5-Ba(2)-O(3)#5	45.6(2)
O(11)-Ba(2)-O(9)#3	171.3(3)	O(24)-Ba(2)-B(9)	57.3(3)
O(1)#3-Ba(2)-O(9)#3	49.0(2)	O(11)-Ba(2)-B(9)	27.0(3)
O(13)-Ba(2)-O(9)#3	64.8(3)	O(1)#3-Ba(2)-B(9)	98.3(3)
O(10)-Ba(2)-O(9)#3	122.2(2)	O(13)-Ba(2)-B(9)	106.7(3)
O(8)#4-Ba(2)-O(9)#3	69.9(2)	O(10)-Ba(2)-B(9)	26.0(3)
O(24)-Ba(2)-O(15)#4	91.0(3)	O(8)#4-Ba(2)-B(9)	142.1(3)
O(11)-Ba(2)-O(15)#4	69.6(2)	O(9)#3-Ba(2)-B(9)	147.2(3)
O(1)#3-Ba(2)-O(15)#4	165.8(3)	O(15)#4-Ba(2)-B(9)	94.0(3)
O(13)-Ba(2)-O(15)#4	97.2(3)	O(7)#5-Ba(2)-B(9)	110.7(3)
O(10)-Ba(2)-O(15)#4	119.6(2)	O(3)#5-Ba(2)-B(9)	109.4(3)

O(24)-Ba(2)-B(8)#4	103.5(3)	O(3)-Ba(3)-O(14)#2	69.5(3)
O(11)-Ba(2)-B(8)#4	93.2(3)	O(19)#3-Ba(3)-O(14)#2	118.8(3)
O(1)#3-Ba(2)-B(8)#4	143.0(3)	O(7)-Ba(3)-O(14)#2	117.8(2)
O(13)-Ba(2)-B(8)#4	89.5(3)	O(17)-Ba(3)-O(10)#6	173.5(3)
O(10)-Ba(2)-B(8)#4	143.6(3)	O(16)#2-Ba(3)-O(10)#6	77.1(3)
O(8)#4-Ba(2)-B(8)#4	24.3(3)	O(6)#3-Ba(3)-O(10)#6	96.4(3)
O(9)#3-Ba(2)-B(8)#4	94.2(3)	O(3)-Ba(3)-O(10)#6	110.5(3)
O(15)#4-Ba(2)-B(8)#4	24.1(3)	O(19)#3-Ba(3)-O(10)#6	74.6(3)
O(7)#5-Ba(2)-B(8)#4	99.4(3)	O(7)-Ba(3)-O(10)#6	115.6(2)
O(3)#5-Ba(2)-B(8)#4	59.3(3)	O(14)#2-Ba(3)-O(10)#6	94.3(3)
B(9)-Ba(2)-B(8)#4	118.0(4)	O(17)-Ba(3)-O(8)#6	139.0(3)
O(17)-Ba(3)-O(16)#2	101.9(3)	O(16)#2-Ba(3)-O(8)#6	94.2(2)
O(17)-Ba(3)-O(6)#3	78.3(3)	O(6)#3-Ba(3)-O(8)#6	126.0(3)
O(16)#2-Ba(3)-O(6)#3	118.1(3)	O(3)-Ba(3)-O(8)#6	63.5(2)
O(17)-Ba(3)-O(3)	75.7(3)	O(19)#3-Ba(3)-O(8)#6	122.0(2)
O(16)#2-Ba(3)-O(3)	118.1(3)	O(7)-Ba(3)-O(8)#6	88.5(2)
O(6)#3-Ba(3)-O(3)	121.5(3)	O(14)#2-Ba(3)-O(8)#6	73.2(3)
O(17)-Ba(3)-O(19)#3	98.9(3)	O(10)#6-Ba(3)-O(8)#6	47.4(2)
O(16)#2-Ba(3)-O(19)#3	70.4(3)	O(17)-Ba(3)-O(9)#7	124.4(3)
O(6)#3-Ba(3)-O(19)#3	49.2(3)	O(16)#2-Ba(3)-O(9)#7	129.9(3)
O(3)-Ba(3)-O(19)#3	170.5(2)	O(6)#3-Ba(3)-O(9)#7	61.8(3)
O(17)-Ba(3)-O(7)	66.6(3)	O(3)-Ba(3)-O(9)#7	93.1(2)
O(16)#2-Ba(3)-O(7)	163.9(3)	O(19)#3-Ba(3)-O(9)#7	83.4(2)
O(6)#3-Ba(3)-O(7)	72.0(3)	O(7)-Ba(3)-O(9)#7	65.4(2)
O(3)-Ba(3)-O(7)	49.6(2)	O(14)#2-Ba(3)-O(9)#7	137.4(3)
O(19)#3-Ba(3)-O(7)	121.2(2)	O(10)#6-Ba(3)-O(9)#7	54.6(2)
O(17)-Ba(3)-O(14)#2	89.7(3)	O(8)#6-Ba(3)-O(9)#7	64.3(2)
O(16)#2-Ba(3)-O(14)#2	48.6(3)	O(17)-Ba(3)-B(10)#3	91.0(3)
O(6)#3-Ba(3)-O(14)#2	160.1(3)	O(16)#2-Ba(3)-B(10)#3	94.4(3)

O(6)#3-Ba(3)-B(10)#3	25.2(3)	O(7)#1-Al(2)-O(20)	115.2(5)
O(3)-Ba(3)-B(10)#3	146.6(3)	O(7)#1-Al(2)-O(14)#2	110.0(5)
O(19)#3-Ba(3)-B(10)#3	24.2(3)	O(20)-Al(2)-O(14)#2	106.6(5)
O(7)-Ba(3)-B(10)#3	97.0(3)	O(7)#1-Al(2)-O(19)	107.2(5)
O(14)#2-Ba(3)-B(10)#3	142.1(3)	O(20)-Al(2)-O(19)	107.6(5)
O(10)#6-Ba(3)-B(10)#3	82.7(3)	O(14)#2-Al(2)-O(19)	110.2(5)
O(8)#6-Ba(3)-B(10)#3	125.4(3)	O(23)-B(1)-O(21)	121.1(12)
O(9)#7-Ba(3)-B(10)#3	69.1(3)	O(23)-B(1)-O(24)	120.8(12)
O(17)-Ba(3)-B(11)#2	97.8(3)	O(21)-B(1)-O(24)	118.0(12)
O(16)#2-Ba(3)-B(11)#2	24.4(3)	O(21)-B(2)-O(22)	122.7(14)
O(6)#3-Ba(3)-B(11)#2	141.4(3)	O(21)-B(2)-O(20)	115.7(14)
O(3)-Ba(3)-B(11)#2	93.7(3)	O(22)-B(2)-O(20)	121.5(13)
O(19)#3-Ba(3)-B(11)#2	94.8(3)	O(5)-B(3)-O(13)	122.6(13)
O(7)-Ba(3)-B(11)#2	141.9(3)	O(5)-B(3)-O(4)	116.4(12)
O(14)#2-Ba(3)-B(11)#2	24.3(3)	O(13)-B(3)-O(4)	121.0(12)
O(10)#6-Ba(3)-B(11)#2	84.0(3)	O(5)-B(3)-Ba(1)#4	60.6(7)
O(8)#6-Ba(3)-B(11)#2	81.9(3)	O(13)-B(3)-Ba(1)#4	164.4(11)
O(9)#7-Ba(3)-B(11)#2	137.6(3)	O(4)-B(3)-Ba(1)#4	57.9(6)
B(10)#3-Ba(3)-B(11)#2	118.7(4)	O(12)-B(4)-O(2)	114.7(13)
O(5)-Al(1)-O(10)#4	119.3(5)	O(12)-B(4)-O(17)	123.6(13)
O(5)-Al(1)-O(15)#8	106.7(5)	O(2)-B(4)-O(17)	121.7(14)
O(10)#4-Al(1)-O(15)#8	109.9(5)	O(12)-B(4)-Ba(1)#9	57.4(7)
O(5)-Al(1)-O(9)#3	106.7(5)	O(2)-B(4)-Ba(1)#9	57.4(7)
O(10)#4-Al(1)-O(9)#3	104.7(5)	O(17)-B(4)-Ba(1)#9	176.9(10)
O(15)#8-Al(1)-O(9)#3	109.2(5)	O(24)-B(5)-O(13)	106.1(11)
O(5)-Al(1)-Ba(3)#5	151.4(3)	O(24)-B(5)-O(18)	113.9(12)
O(10)#4-Al(1)-Ba(3)#5	52.0(3)	O(13)-B(5)-O(18)	109.3(11)
O(15)#8-Al(1)-Ba(3)#5	101.6(4)	O(24)-B(5)-O(12)	110.7(11)
O(9)#3-Al(1)-Ba(3)#5	58.8(3)	O(13)-B(5)-O(12)	108.9(11)

O(18)-B(5)-O(12)	108.0(11)	O(23)-B(9)-O(11)	108.3(11)
O(24)-B(5)-Ba(2)	51.2(6)	O(10)-B(9)-O(8)	109.2(11)
O(13)-B(5)-Ba(2)	54.9(6)	O(23)-B(9)-O(8)	106.9(10)
O(18)-B(5)-Ba(2)	127.6(9)	O(11)-B(9)-O(8)	107.4(10)
O(12)-B(5)-Ba(2)	124.4(8)	O(10)-B(9)-Ba(2)	59.5(6)
O(18)-B(6)-O(4)	110.8(10)	O(23)-B(9)-Ba(2)	104.9(8)
O(18)-B(6)-O(22)#3	111.7(11)	O(11)-B(9)-Ba(2)	57.1(5)
O(4)-B(6)-O(22)#3	113.7(11)	O(8)-B(9)-Ba(2)	147.8(9)
O(18)-B(6)-O(17)	106.2(11)	O(10)-B(9)-Ba(3)#11	58.1(7)
O(4)-B(6)-O(17)	108.2(11)	O(23)-B(9)-Ba(3)#11	159.0(9)
O(22)#3-B(6)-O(17)	105.8(10)	O(11)-B(9)-Ba(3)#11	92.4(8)
O(18)-B(6)-Ba(1)	49.8(6)	O(8)-B(9)-Ba(3)#11	61.8(6)
O(4)-B(6)-Ba(1)	145.8(9)	Ba(2)-B(9)-Ba(3)#11	89.1(4)
O(22)#3-B(6)-Ba(1)	64.3(6)	O(19)-B(10)-O(3)	124.1(12)
O(17)-B(6)-Ba(1)	104.9(7)	O(19)-B(10)-O(6)	117.1(11)
O(11)-B(7)-O(9)	122.0(11)	O(3)-B(10)-O(6)	118.8(12)
O(11)-B(7)-O(1)	120.4(12)	O(19)-B(10)-Ba(3)#10	59.6(6)
O(9)-B(7)-O(1)	117.6(11)	O(3)-B(10)-Ba(3)#10	170.8(9)
O(11)-B(7)-Ba(2)#10	174.4(9)	O(6)-B(10)-Ba(3)#10	58.3(6)
O(9)-B(7)-Ba(2)#10	61.9(6)	O(14)-B(11)-O(16)	119.3(12)
O(1)-B(7)-Ba(2)#10	55.9(6)	O(14)-B(11)-O(6)	121.0(12)
O(15)-B(8)-O(8)	121.1(11)	O(16)-B(11)-O(6)	119.7(11)
O(15)-B(8)-O(1)	121.7(11)	O(14)-B(11)-Ba(3)#9	62.8(7)
O(8)-B(8)-O(1)	117.1(11)	O(16)-B(11)-Ba(3)#9	56.8(6)
O(15)-B(8)-Ba(2)#1	62.5(7)	O(6)-B(11)-Ba(3)#9	171.8(10)
O(8)-B(8)-Ba(2)#1	58.9(6)	O(7)-B(12)-O(2)	117.0(12)
O(1)-B(8)-Ba(2)#1	174.9(9)	O(7)-B(12)-O(16)	110.7(10)
O(10)-B(9)-O(23)	115.7(11)	O(2)-B(12)-O(16)	107.3(11)
O(10)-B(9)-O(11)	109.0(10)	O(7)-B(12)-O(3)	107.0(11)

O(2)-B(12)-O(3)	107.5(10)	B(10)-O(6)-B(11)	121.1(10)
O(16)-B(12)-O(3)	106.9(10)	B(10)-O(6)-Ba(3)#10	96.5(7)
O(7)-B(12)-Ba(3)	59.2(6)	B(11)-O(6)-Ba(3)#10	140.3(8)
O(2)-B(12)-Ba(3)	100.0(8)	B(12)-O(7)-Al(2)#4	119.9(8)
O(16)-B(12)-Ba(3)	152.3(9)	B(12)-O(7)-Ba(3)	95.2(7)
O(3)-B(12)-Ba(3)	58.5(6)	Al(2)#4-O(7)-Ba(3)	128.3(4)
O(7)-B(12)-Ba(2)#12	63.8(7)	B(12)-O(7)-Ba(2)#12	90.8(7)
O(2)-B(12)-Ba(2)#12	175.9(9)	Al(2)#4-O(7)-Ba(2)#12	123.2(4)
O(16)-B(12)-Ba(2)#12	75.7(7)	Ba(3)-O(7)-Ba(2)#12	89.9(2)
O(3)-B(12)-Ba(2)#12	68.6(6)	B(8)-O(8)-B(9)	116.7(11)
Ba(3)-B(12)-Ba(2)#12	76.9(3)	B(8)-O(8)-Ba(2)#1	96.8(7)
B(7)-O(1)-B(8)	121.2(11)	B(9)-O(8)-Ba(2)#1	135.7(7)
B(7)-O(1)-Ba(2)#10	99.5(8)	B(8)-O(8)-Ba(3)#11	126.8(8)
B(8)-O(1)-Ba(2)#10	139.3(8)	B(9)-O(8)-Ba(3)#11	92.2(7)
B(4)-O(2)-B(12)	130.0(11)	Ba(2)#1-O(8)-Ba(3)#11	89.4(2)
B(4)-O(2)-Ba(1)#9	97.9(9)	B(7)-O(9)-Al(1)#10	123.2(8)
B(12)-O(2)-Ba(1)#9	131.5(8)	B(7)-O(9)-Ba(2)#10	93.5(7)
B(10)-O(3)-B(12)	116.8(10)	Al(1)#10-O(9)-Ba(2)#10	120.8(4)
B(10)-O(3)-Ba(3)	142.3(8)	B(7)-O(9)-Ba(3)#13	137.7(8)
B(12)-O(3)-Ba(3)	94.1(7)	Al(1)#10-O(9)-Ba(3)#13	92.1(3)
B(10)-O(3)-Ba(2)#12	114.7(8)	Ba(2)#10-O(9)-Ba(3)#13	85.8(2)
B(12)-O(3)-Ba(2)#12	84.4(7)	B(9)-O(10)-Al(1)#1	124.0(8)
Ba(3)-O(3)-Ba(2)#12	87.8(2)	B(9)-O(10)-Ba(2)	94.5(7)
B(3)-O(4)-B(6)	121.7(11)	Al(1)#1-O(10)-Ba(2)	127.5(4)
B(3)-O(4)-Ba(1)#4	95.9(7)	B(9)-O(10)-Ba(3)#11	97.4(8)
B(6)-O(4)-Ba(1)#4	142.3(8)	Al(1)#1-O(10)-Ba(3)#11	100.1(4)
B(3)-O(5)-Al(1)	128.5(9)	Ba(2)-O(10)-Ba(3)#11	109.4(3)
B(3)-O(5)-Ba(1)#4	93.5(8)	B(7)-O(11)-B(9)	117.9(10)
Al(1)-O(5)-Ba(1)#4	138.0(5)	B(7)-O(11)-Ba(2)	144.8(8)

B(9)-O(11)-Ba(2)	95.9(7)	B(4)-O(17)-B(6)	112.3(10)
B(4)-O(12)-B(5)	121.6(11)	B(4)-O(17)-Ba(3)	123.4(8)
B(4)-O(12)-Ba(1)#9	98.1(8)	B(6)-O(17)-Ba(3)	122.1(7)
B(5)-O(12)-Ba(1)#9	138.0(8)	B(6)-O(18)-B(5)	110.0(10)
B(3)-O(13)-B(5)	114.4(11)	B(6)-O(18)-Ba(1)	104.6(7)
B(3)-O(13)-Ba(2)	144.2(9)	B(5)-O(18)-Ba(1)	143.1(8)
B(5)-O(13)-Ba(2)	99.4(7)	B(10)-O(19)-Al(2)	128.5(9)
B(11)-O(14)-Al(2)#9	136.7(9)	B(10)-O(19)-Ba(3)#10	96.2(7)
B(11)-O(14)-Ba(3)#9	92.9(8)	Al(2)-O(19)-Ba(3)#10	126.3(4)
Al(2)#9-O(14)-Ba(3)#9	129.8(4)	B(2)-O(20)-Al(2)	135.3(10)
B(8)-O(15)-Al(1)#14	140.2(9)	B(2)-O(21)-B(1)	151.6(13)
B(8)-O(15)-Ba(2)#1	93.4(7)	B(2)-O(22)-B(6)#10	132.2(11)
Al(1)#14-O(15)-Ba(2)#1	126.2(4)	B(2)-O(22)-Ba(1)#10	111.5(8)
B(11)-O(16)-B(12)	117.1(10)	B(6)#10-O(22)-Ba(1)#10	89.1(7)
B(11)-O(16)-Ba(3)#9	98.8(7)	B(1)-O(23)-B(9)	125.4(10)
B(12)-O(16)-Ba(3)#9	142.0(7)	B(1)-O(24)-B(5)	127.1(11)
B(11)-O(16)-Ba(2)#12	110.9(8)	B(1)-O(24)-Ba(2)	128.7(8)
B(12)-O(16)-Ba(2)#12	78.2(7)	B(5)-O(24)-Ba(2)	104.2(7)
Ba(3)#9-O(16)-Ba(2)#12	101.0(3)		

Symmetry transformations used to generate equivalent atoms:

#1 x+1/2,y+1/2,z #2 x-1/2,y+1/2,z #3 x-1,y,z
#4 x-1/2,y-1/2,z #5 x,-y+1,z+1/2 #6 x-1/2,-y+3/2,z-1/2
#7 x-1,-y+1,z-1/2 #8 x-3/2,y-1/2,z #9 x+1/2,y-1/2,z
#10 x+1,y,z #11 x+1/2,-y+3/2,z+1/2 #12 x,-y+1,z-1/2
#13 x+1,-y+1,z+1/2 #14 x+3/2,y+1/2,z

Table S4. Comparison of the properties of $Ba_3Al_2B_{12}O_{24}$ with the reported UV NLO aluminoborates.

**Compounds**	**Space group**	**SHG (× KDP)**	**UV cutoff edge (nm)**	**Ref.**
$RbAlB_3O_6F$	$Pna2_1$	0.2	< 200	[8]
$Cs_2Al_2B_6O_{13}$	$P6_3$	0.5	185	[9]
$K_2Al_2B_2O_7$	$P321$	0.9	180	[10]
$Rb_3Al_3B_3O_{10}F$	$P31c$	1.2	< 200	[11]
$Rb_3Ba_3Li_2Al_4B_6O_{20}F$	$P\bar{6}2c$	1.5	198	[12]
$K_3Ba_3Li_2Al_4B_6O_{20}F$	$P\bar{6}2c$	1.5	190	[13]
$K_3Sr_3Li_2Al_4B_6O_{20}F$	$R32$	1.7	190	[14]
$BaAl_2B_2O_7$	$R32$	1.7	200	[15]
β-$Rb_2Al_2B_2O_7$	$P321$	2	< 200	[16]
$BaAlBO_3F_2$	$P\bar{6}2c$	2	165	[17]
$CsAlB_3O_6F$	$Pna2_1$	2	< 190	[18]
$Ba_3Al_2B_{12}O_{24}$	Cc	2.7	< 190	This work

Table S5. The sum of the dipole moments and the normalized dipole moment of all corresponding units [AlO_4], [B_2O_5], [B_4O_9], and [B_3O_7] in the unit cell of $Ba_3Al_2B_{12}O_{24}$.

Polar unit	*X*-axis	*Y*-axis	*Z*-axis	Magnitude	
				debye (D)	esu•cm/Å^3
[AlO_4]	-3.34	0	3.97	5.24	0.003
[B_2O_5]	-4.30	0	-2.57	4.97	0.003
[B_4O_9]	6.96	0	-8.39	10.99	0.006
[B_3O_7]	19.30	0	-2.58	19.52	0.010
B-O Total	21.96	0	-13.54	26.01	0.013

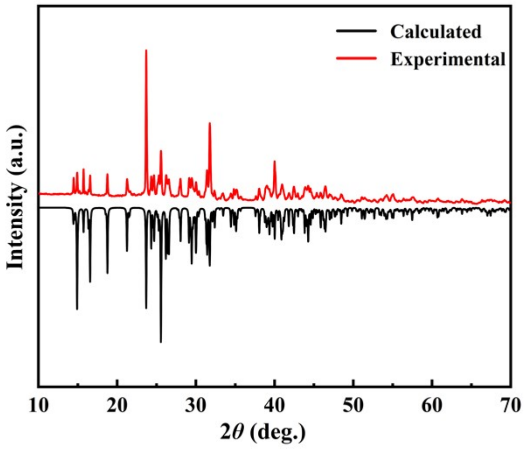

Figure S1. Calculated and experimental PXRD patterns of $Ba_3Al_2B_{12}O_{24}$.

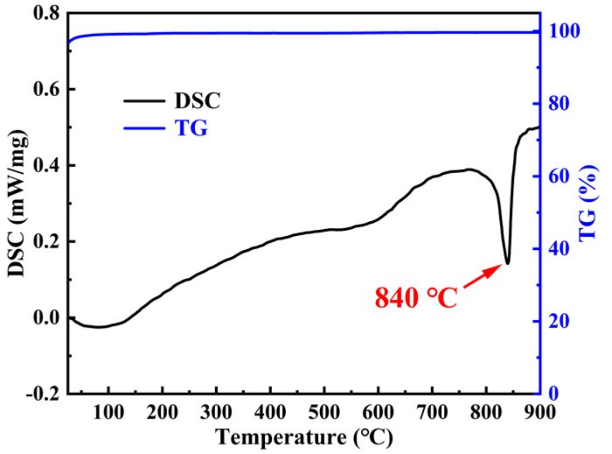

Figure S2. The TG/DSC curves for $Ba_3Al_2B_{12}O_{24}$.

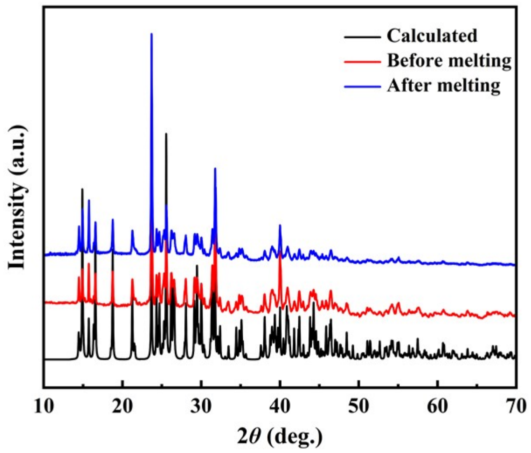

Figure S3. The PXRD of $Ba_3Al_2B_{12}O_{24}$ before and after melting (850 °C).

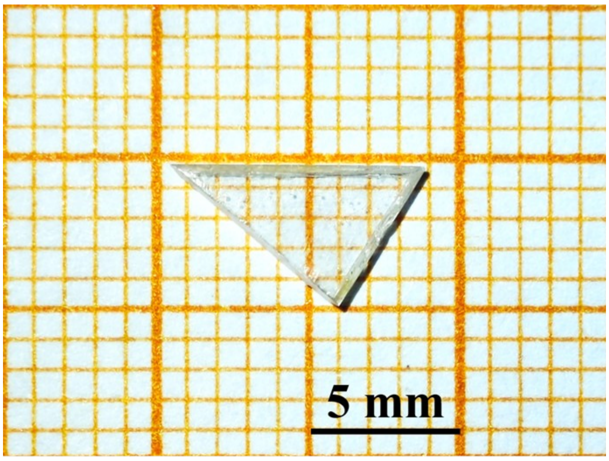

Figure S4. $Ba_3Al_2B_{12}O_{24}$ with a size of 9 × 5 × 2 mm^3.

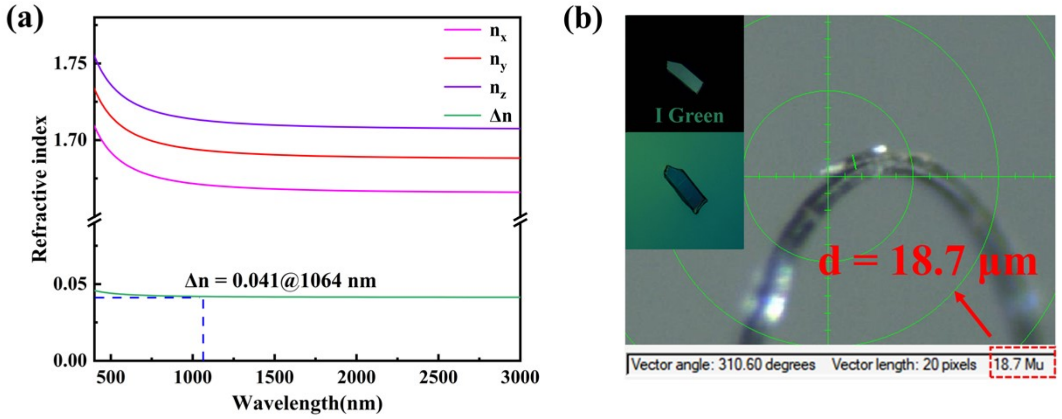

Figure S5. (a) The calculated birefringence of $Ba_3Al_2B_{12}O_{24}$. (b) Birefringence (Δn) measurement of $Ba_3Al_2B_{12}O_{24}$.

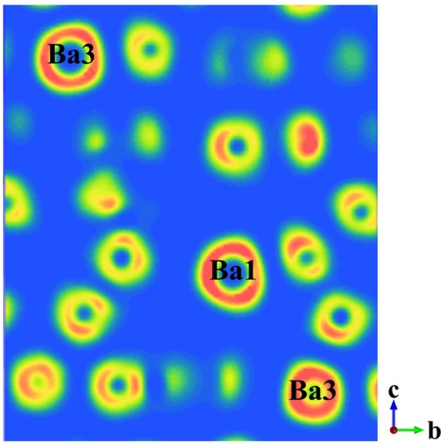

Figure S6. The sliced-plane of electron localization function (ELF) of $Ba_3Al_2B_{12}O_{24}$ in the *bc*-plane.

References

[1] L. Krause, R. Herbst-Irmer, G. Sheldrick and D. Stalke, *J. Appl. Crystallogr.*, 2015, **48**, 3–10.

[2] G. Sheldrick, *Acta Crystallogr. A Found. Adv.*, 2015, **71**, 3–8.

[3] A. Spek, *J. Appl. Crystallogr.*, 2003, **36**, 7–13.

[4] S. Kurtz and T. Perry, *J. Appl. Phys.*, 1968, **39**, 3798–3813.

[5] S. Clark, M. Segall, C. Pickard, P. Hasnip, M. Probert, K. Refson and M. Payne, *Z. Kristallogr. - Cryst. Mater.*, 2005, **220**, 567–570.

[6] J. Perdew, K. Burke and M. Ernzerhof, *Phys. Rev. Lett.*, 1996, **77**, 3865–3868.

[7] J. Lin, A. Qteish, M. Payne and V. Heine, *Phys. Rev. B*, 1993, **47**, 4174–4180.

[8] H. Liu, B. Zhang, L. Li and Y. Wang, *ACS Appl. Mater. Interfaces*, 2021, **13**, 30853–30860.

[9] Z. Fang, X. Jiang, M. Duan, Z. Hou, C. Tang, M. Xia, L. Liu, Z. Lin, F. Fan, L. Bai and C. Chen, *Chem. - Eur. J.*, 2018, **24**, 7856–7860.

[10] N. Ye, W. Zeng, J. Jiang, B. Wu, C. Chen, B. Feng and X. Zhang, *J. Opt. Soc. Am. B*, 2000, **17**, 764–768.

[11] S. Zhao, P. Gong, S. Luo, S. Liu, L. Li, M. Asghar, T. Khan, M. Hong, Z. Lin and J. Luo, *J. Am. Chem. Soc.*, 2015, **137**, 2207–2210.

[12] H. Yu, J. Young, H. Wu, W. Zhang, J. Rondinelli and P. Halasyamani, *Adv. Opt. Mater.*, 2017, **5**, 1700840.

[13] S. Zhao, L. Kang, Y. Shen, X. Wang, M. Asghar, Z. Lin, Y. Xu, S. Zeng, M. Hong and J. Luo, *J. Am. Chem. Soc.*, 2016, **138**, 2961–2964.

[14] H. Wu, H. Yu, S. Pan and P. Halasyamani, *Inorg. Chem.*, 2017, **56**, 8755–8758.

[15] Z. Lin, Z. Wang, C. Chen, S. Chen and M. Lee, *J. Appl. Phys.*, 2003, **93**, 9717–9723.

[16] T. Tran, N. Koocher, J. Rondinelli and P. Halasyamani, *Angew. Chem.*, 2017, **129**, 3015–3019.

[17] Z. Hu, Y. Yue, X. Chen, J. Yao, J. Wang and Z. Lin, *Solid State Sci.*, 2011, **13**, 875–878.

[18] H. Liu, Y. Wang, B. Zhang, Z. Yang and S. Pan, *Chem. Sci.*, 2020, **11**, 694–698.
